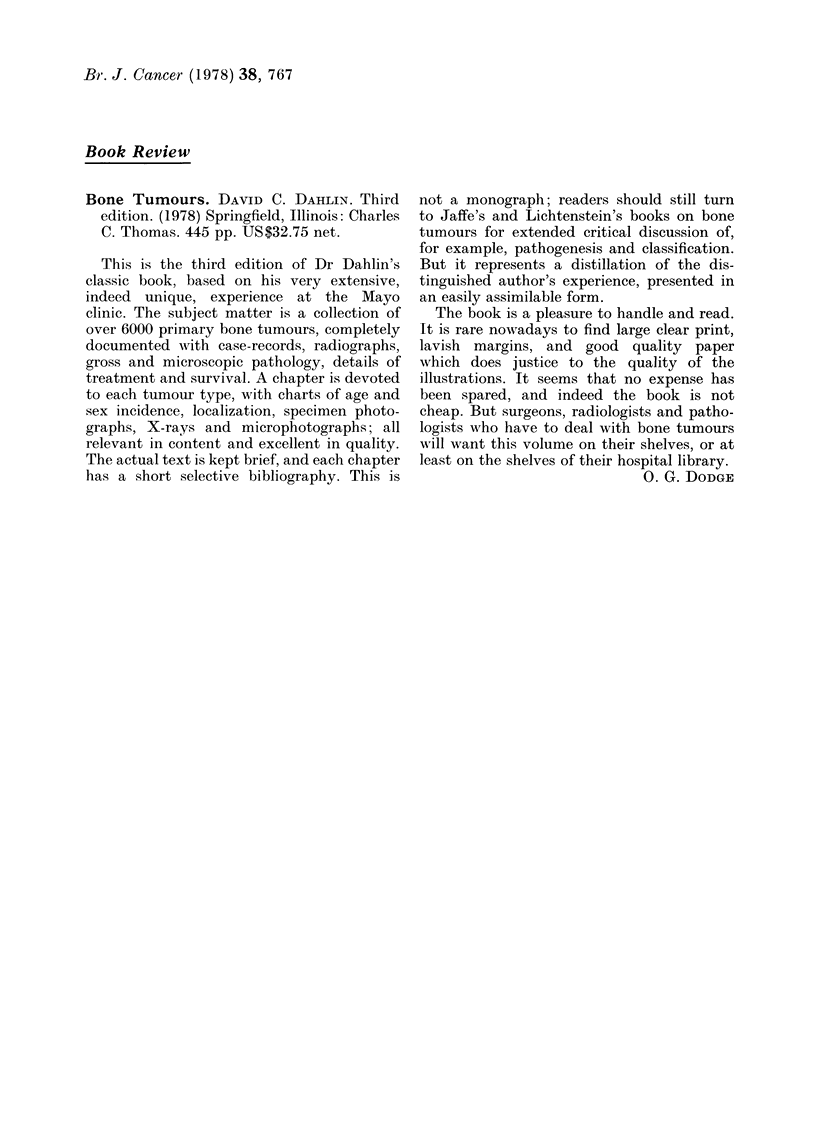# Bone Tumours

**Published:** 1978-12

**Authors:** O. G. Dodge


*Br. J. Cancer* (1978) **38**, 767

### *Book Review*

**Bone Tumours.** DAVID C. DAHLIN. Third edition. (1978) Springfield, Illinois: Charles C. Thomas. 445 pp. US$32.75 net.

This is the third edition of Dr Dahlin's classic book, based on his very extensive, indeed unique, experience at the Mayo clinic. The subject matter is a collection of over 6000 primary bone tumours, completely documented with case-records, radiographs, gross and microscopic pathology, details of treatment and survival. A chapter is devoted to each tumour type, with charts of age and sex incidence, localization, specimen photographs, X-rays and microphotographs; all relevant in content and excellent in quality. The actual text is kept brief, and each chapter has a short selective bibliography. This is not a monograph; readers should still turn to Jaffe's and Lichtenstein's books on bone tumours for extended critical discussion of, for example, pathogenesis and classification. But it represents a distillation of the distinguished author's experience, presented in an easily assimilable form.

The book is a pleasure to handle and read. It is rare nowadays to find large clear print, lavish margins, and good quality paper which does justice to the quality of the illustrations. It seems that no expense has been spared, and indeed the book is not cheap. But surgeons, radiologists and pathologists who have to deal with bone tumours will want this volume on their shelves, or at least on the shelves of their hospital library.

O. G. DODGE